# Transverse vs. median laparotomy in peritonitis and staged lavage: a single center case series

**DOI:** 10.3205/000283

**Published:** 2020-09-14

**Authors:** Sven Petersen, Alice Deder, Axel Prause, Christopher Pohland, Dominik Richter, Thomas Mansfeld, Gero Puhl

**Affiliations:** 1 Department of General, Visceral and Vascular Surgery, Asklepios Hospital Altona, Hamburg, Germany; 2 Department of Anesthesia, Intensive Care & Emergency, Asklepios Hospital Altona, Hamburg, Germany; 3 Department of Surgery, Westklinikum Hamburg Rissen, Hamburg, Germany

**Keywords:** peritonitis, staged lavage, wound healing

## Abstract

**Background:** Staged lavage was first introduced in the 1970s and now serves as a therapeutic option for septic patients with peritonitis. A central aspect of this treatment concept is leaving the abdomen open after a wide incision. To evaluate the influence of transverse vs. median access to the abdomen in staged lavage, data from the authors’ patients were analyzed.

**Methods:** To evaluate patients with peritonitis, prospective intensive care data were examined together with data on the surgical details. The main aspects covered here were the surgical details of the lavage (namely, transverse vs. median laparotomy), number of lavages, fascia closure, wound-healing disorders, and observed lethality, in combination with the preoperatively evaluated SAPS-II score, expected hospital lethality, patient age, and the Mannheim Peritonitis Index.

**Results:** Between January 2008 and December 2018, 522 patients were treated with open abdomen and staged lavage. The mean age of the patients was 66.0 years (standard deviation (SD) 15.9 years). A median incision was used in 140 cases, and transverse laparotomy was performed in 382. The mean SAPS-II score was 46.5 (SD 15.7), expected lethality was 39.6% (SD 26.3%), and observed lethality was 19.9%. On average, two lavages were performed after the index operation. Transverse incision was significantly less likely to cause wound-healing disorder (p=0.03), and fascial dehiscence was observed less frequently in the transverse laparotomies group than in median incisions in the statistical trend (p=0.06).

**Conclusion:** In summary, staged lavage reduced expected lethality in patients with peritonitis. Transverse incision caused wound-healing disorders and fascial dehiscence less often. Therefore, the indication for transverse laparotomy should be generous, as this form of treatment is advantageous in case of peritonitis.

## Zusammenfassung

**Hintergrund:** Die sogenannte Etappenlavage wurde erstmals in den 1970er Jahren eingeführt und dient bis heute als therapeutische Option für septische Patienten mit Peritonitis. Ein zentraler Aspekt dieses Behandlungskonzepts ist es, den Bauch nach einer weiten Laparotomie offen zu belassen. Um den Einfluss der queren vs. medianen Laparotomie bei der Etappenlavage zu bewerten, wurden eigene Daten analysiert.

**Methoden:** Zur Beurteilung von Patienten mit Peritonitis wurden prospektive Intensivmedizindaten zusammen mit Daten zu chirurgischen Details untersucht. Die wichtigsten Aspekte waren die chirurgischen Details der Lavage (nämlich quere vs. mediane Laparotomie), Anzahl der Lavagen, Faszienverschluss, Wundheilungsstörungen und beobachtete Letalität, in Kombination mit dem präoperativ ausgewerteten SAPS-II-Score, der erwarteten Krankenhausmortalität, dem Patientenalter und dem Mannheimer Peritonitis-Index.

**Ergebnisse:** Zwischen Januar 2008 und Dezember 2018 wurden 522 Patienten mit offenem Bauch und Etappenlavage behandelt. Das Durchschnittsalter der Patienten betrug 66,0 Jahre (Standardabweichung (SD) 15,9 Jahre). In 140 Fällen wurde ein medianer Schnitt verwendet, und die quere Laparotomie wurde in 382 Fällen durchgeführt. Der durchschnittliche SAPS-II-Score lag bei 46,5 (SD 15,7), die erwartete Letalität lag bei 39,6% (SD 26,3%) und die beobachtete Letalität bei 19,9%. Im Durchschnitt wurden nach der Indexoperation zwei Lavages durchgeführt. Die quere Laparotomie führte dabei signifikant seltener zu Wundheilungsstörungen (p=0,03), und die fasziale Dehiszenz wurde in der Gruppe der queren Laparotomien ebenfalls seltener beobachtet als bei medianen Einschnitten im statistischen Trend (p=0,06).

**Schlussfolgerung:** Zusammenfassend reduzierte Etappenlavage die erwartete Letalität bei Patienten mit Peritonitis. Die quere Laparotomie verursachte seltener Wundheilungsstörungen und Fasziendehiszenzen. Daher sollte die Indikation für die transversale Laparotomie großzügig sein, da diese Behandlungsform bei Peritonitis vorteilhaft ist.

## Introduction

Peritonitis is a life-threatening condition, associated with high rates of organ failure and mortality. Surgical intervention is essential for the treatment of severe peritonitis. During surgery, the most important steps are the eradication of the infection focus and the cleaning of the abdominal cavity. For this purpose, a range of surgical approaches have been developed in recent decades [1], [2], [3]. One treatment option is to perform explorative laparotomy to eliminate the cause of infection. After this is done, the abdomen may be left open to allow the intestine to swell without impairing the abdominal compartment. To eliminate infection remnants from the peritoneal cavity, repeated re-exploration of the abdomen is recommended. This repeated exploration and washing of the peritoneal surface is called staged lavage or open abdomen treatment (OAT). Staged lavage therapy (Etappenlavage) was first introduced in the early 1970s at several surgical institutions that specialized in peritonitis treatment [4], [5]. In Leiden, Penninckx et al. [6] developed a concept of reoperation at intervals of 2 days, and at Altona Hospital, the practice was for the peritoneal cavity to be cleaned daily [6], [7].

An essential aspect of staged lavage treatment is the performance of laparotomy at the moment when peritonitis is occurring. If the abdominal wall remains open for a longer time, the abdominal muscles may retract, causing difficulty in closing the abdominal wall. The longer the lavage lasts, the higher the risk that the abdominal wall will remain open after the treatment is complete.

There is some evidence that transverse abdominal wall incision is superior to median laparotomy in terms of the closure rate at the end of staged lavage and the complication rate.

This study evaluated the influence of median or transverse incision.

## Methods

Data of patients who underwent staged lavage in the period from January 2008 to December 2018 were analyzed. Ethical approval for retrospective analysis was provided by the Ethics Committee of Hamburg Medical Association (Hamburger Ärztekammer), Germany, register no. #WF072/20. The following variables were recorded: age at admission, SAPS-II score, Mannheim Peritonitis Index, number of staged lavages, hours of ventilation, incision type, median vs. transverse incision during staged lavage, fascia closure at the end of the staged lavage, presence of a wound-healing disorder, fascial dehiscence, postoperative complications, and mortality [8], [9], [10].

The intervention of staged lavage has been described previously and is now standard procedure [7]. In brief, patients undergo explorative laparotomy if peritonitis is suspected. The vast majority of patients acquire peritonitis as a consequence of prior abdominal procedures. Usually, laparotomy is done using the laparotomy site of the initial procedure. The abdominal cavity is left open, the small bowel is covered in a Vi-Drape^®^ intestine bag as visceral protection layer (Cardinal Health GmbH, Norderstedt, Germany), and Parietex^®^ mesh (Medtronic GmbH, Meerbusch, Germany) is sutured into the dorsal aspect of the rectus muscle. In our patients, in the days following the initial procedure, the abdomen was reopened daily and an abdominal lavage was conducted. For this purpose, the Parietex^®^ mesh was incised in the middle. As soon as the swelling of the abdomen decreased, the inserted mesh was reduced from the middle to achieve reapproximation of the fascial edges. No vacuum was used.

In the present study, type of incision, transverse or median, was recorded at the time of laparotomy. Usually, the type of incision was selected according to the incision that had previously been performed. When no previous abdominal incision or laparoscopic trocar incisions were known or observed, a transverse incision was used.

Wound-healing disorders were taken from the charts and were defined as any evidence of cutaneous wound infection with or without germ detection in bacteriology as clinical sign of wound redness or leakage of pus. In addition, any necessity for removing stitches earlier than planned was also considered as a wound-healing disorder.

Any evidence of reopening of the fascia after staged lavage was defined as fascial dehiscence. In the clinical suspicion of fascial dehiscence, for example due to a gaping wound or by an increased secretion of seroma, a clinical examination with a sterile glove was carried out to exclude interruption of fascial continuity. Complications were scored using the Clavien-Dindo classification [8].

Data on the severity of the disease, as determined by the SAPS-II score, were collected prospectively upon admission to the intensive care unit (ICU). The surgical results were determined retrospectively.

In order to evaluate whether the results changed during the years, the results from 2008 to 2012 were compared to those from 2013 to 2018. In this subgroup analysis, the major variables such as SAPS-II score, MPI score and type of incisions, as well as the results: days in ICU, mortality and complication rate were included.

ICU data revealed that 600 patients had undergone staged lavage from 2008 to 2018. From this group, a total of 78 patients (13.0%) were excluded. Reasons for exclusion were uncommon laparotomy or incomplete data in 21 cases (3.5%), and that the fascia could not be closed at the end of staged lavage in an additional 57 patients (9.5%).

Statistical analyses were performed using the SPSS 26.0 software package (IBM Inc., USA). The Pearson chi-square test was used to compare the incidence of variables, and the t-test was used to compare the means for the groups’ median and transverse laparotomy. Survival analyses were performed using Kaplan-Meier survival analysis and curves. Variables with p-values less than 0.05 were considered significant.

## Results

A total of 522 patients with complete data and closure at the end of staged lavage between January 2008 and December 2018 were evaluated.

In 140 cases (26.8%), the abdomen was subject to a median laparotomy, and in 382 cases (73.2%), the laparotomy was transverse. On average, two lavages (standard deviation (SD) 1.4) were performed after the index operation. The patients were in the ICU for an average of 15.5 days (SD 18.1 days) and had to be ventilated for an average of 281 h (SD 335 h), with an observed lethality of 19.9%.

**Figure 1:**
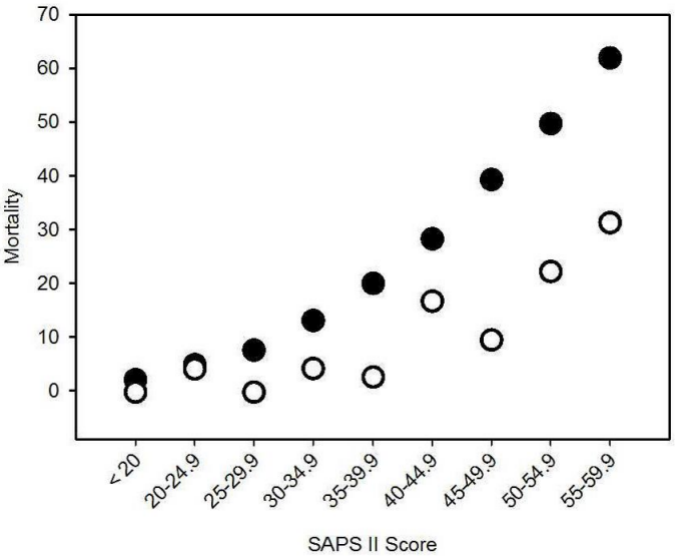
Calculated (dots) and observed (circles) mortality in relation to SAPS-II score

Patients were 66.0 years old on average (SD 15.3) and were in the ICU for 15.5 days (SD 18.1). The average SAPS-II score was 46.5 (SD 15.8), and on average, patients were ventilated for 278 h (SD 335 h). The average Mannheim Peritonitis Index value was 21 (SD 9.8). During the observation period, 104 patients died. Expected lethality resulting from the SAPS-II score was 39.6% (SD 26.3%), compared to an observed lethality of 19.9%. The higher the estimated SAPS-II score, the greater the difference between estimated lethality and observed lethality (Figure 1 (Fig. 1)).

No significant differences were found between the two groups of transverse and median laparotomy in the patient variables (Table 1 (Tab. 1)).

**Figure 2:**
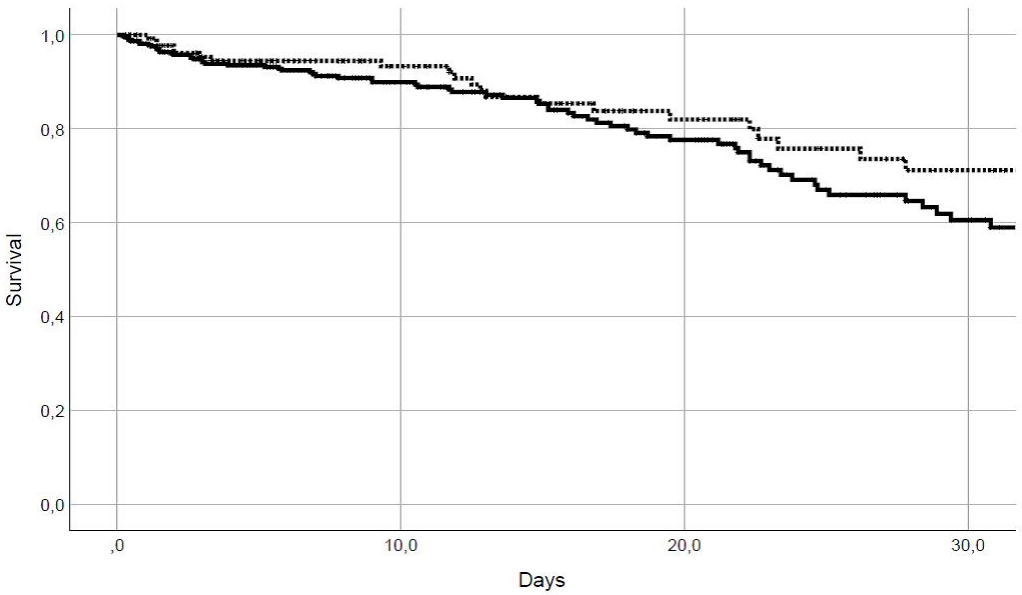
Kaplan Meier survival curve; n=140 patients with median laparotomy (dashed line) versus 382 patients with transverse laparotomy (continuous line); log rank, p=0.16

No differences were found in the Kaplan-Meier curves of survival between the groups with transverse and median incisions (p=0.16) (Figure 2 (Fig. 2)).

Data on patient outcomes are shown in Table 2 (Tab. 2). The total numbers of complications in the groups with transverse and median laparotomy are comparable in terms of Clavien-Dindo classifications for postoperative complications.

However, the group of patients with transverse incisions was significantly less likely to have wound-healing disorders (p=0.03). Fascial dehiscence was also observed less frequently in the cross-laparotomy group (p=0.06).

Concerning the question whether patient characteristics and results changed during the time from 2008 to 2012 (n=298) compared to the period from 2013 to 2018 (n=224), the results revealed no differences with regard to patient characteristics. From 2008 to 2012, the SAPS-II score was 45.5 (SD 15.5) and the MPI 21.1 (9.9), vs. 47.9 (SD 16.0) (p=0.09) and 20.9 (SD 9.6) (p=0.79) in the later timeframe. The ratio of incision was 78 median vs. 220 transverse compared to 62 vs. 162 (p=0.70). According to the results, the stay in ICU was 16.0 days (SD 17.2 days) in the earlier years and 14.7 days (SD 19.3 days) (p=0.40) in the years since 2013. The mortality rate was comparable in both periods 0.20 (SD 0.40) vs. 0.20 (SD 0.39) (p=0.89). In the previous period of time, the rate of wound-healing disorders was 77/298 vs. 52/224 (p=0.49) after 2013. Failure of fascial closure increased from 14/298 in the timeframe until 2012 to 21/203 in the later period (p=0.03).

## Discussion

The staged lavage procedure was established in the 1970s and continues to serve as a crucial treatment option for patients suffering severe sepsis and peritonitis [11], [12]. This surgical approach enables repeated access to the peritoneal cavity to eliminate the remnants of peritonitis and allows the edematous swelling of the intestine. Indication for OAT is a wide range of abdominal conditions from peritonitis associated with mesenteric ischemia to secondary peritonitis due to anastomotic leakage [13], [14]. Consequently, the rate of complications in OAT is substantial [3]. One major consequence of staged lavage is the difficulty of closing the abdominal wall at the end of the lavage [15]. Once the surgical procedure and repeated lavage treatment is complete, the abdominal cavity may remain open or may not be completely closed in a large number of cases [15], [16]. In addition, wound-healing disorders could occur, and this may keep the patient in the hospital for a very long time. Probably the most serious complication is the so-called laparostoma with fascial dehiscence and the danger of a small bowel fistula [15], [17].

To evaluate the influence of access to the abdomen in staged lavage, data from the authors’ patients were analyzed. Analyses of these data showed fewer wound-healing disorders in transverse incisions and a trend toward lower fascial dehiscence in transverse access to the abdomen.

The data presented here describe a select cohort of vitally endangered patients with peritonitis. The results were influenced by a number of crucial factors and therefore are only partially interpretable. First, the patients included in this study were seriously ill, as indicated by the high values for the SAPS-II score and the Mannheim Peritonitis Index. Although the expected lethality as calculated by the SAPS-II score was much higher than the actually observed lethality, an unaccounted-for influence on the results cannot be ruled out. However, Kaplan-Meier survival analyses (Figure 2 (Fig. 2)) indicated no influence of survival on the results.

Furthermore, the fact that patients were taken from several other institutions and departments may have had an influence that cannot be completely ruled out. In other words: there is not always a choice of how to incise in patients with secondary peritonitis since it is most appropriate to use the previous incision. In order to provide transparency for this retrospective analysis, the most important variables are presented in the results section.

Treating peritonitis still means dealing with vitally endangered patients, and therefore it continues to be associated with substantial in-hospital mortality. The mortality rate was close to 20% in the observed group, which is in good agreement with recent results from other working groups that use staged lavage or open abdomen treatment [18], [19]. The estimated mortality calculated from the SAPS-II score was much higher than the observed mortality. This supports the belief that staged lavage reduces mortality considerably [20]. In addition, there was a greater reduction in the mortality rate in the group with a higher SAPS-II score.

A range of aspects must be considered when applying the surgical procedure of staged lavage, and this makes data matching very difficult. However, one advantage of this analysis is the relatively consistent schedule of reoperation and the details of temporary abdominal closure in a large cohort.

One major aspect of staged lavage is the question of how to incise the abdomen. However, it should be noted that usually staged lavage is not a part of the plan for a surgical procedure in the abdomen. However, the potential consequences of a surgical procedure must be taken into account, and the incision therefore needs to be done in such a way that potential complications can be treated under optimal settings.

When the abdominal cavity must be explored due to peritonitis, the surgeon most often follows the incision of the prior surgical procedure. This was also the case in the data presented here, where the older incision was used for re-exploration. Nowadays, this is often easier because the majority of abdominal procedures, particularly for colorectal surgery, are performed laparoscopically, and no prior incision exists. This means that exploration can also be done laparoscopically, and the incision can be performed as necessary.

Fascia closure is a major problem in peritonitis treatment. Atema et al. published an overview indicating that only 50% of patients receive fascia closure at the end of treatment [15]. The data presented here show statistical trends of a lower rate of wound-healing disorders and a lower rate of fascial dehiscence in the transverse incision group. These results were independent of other patient variables (Table 1 (Tab. 1)).

The possibility that transverse incision is advantageous has been recognized for a long time [21]. For example, it has been shown that transverse incision causes less pain in upper GI surgery [22], [23]. Although no one at present would perform routine gallbladder removal by transverse incision, the published data show that transverse incision leads to less pain after surgery.

Many working groups currently consider treatment with the combination of continuous vacuum therapy together with the visceral protection layer and fascial traction to be standard [24]. The idea of this treatment option is to provide fascia approximation as the final outcome. Although the results in this retrospective analysis need to be interpreted with caution, the primary fascia closure rate was close to 90%, and the final closure rate was 84%. In contrast, Willms et al. [19] found a primary closure rate of 79% and Rasilainen et al. [25] one of 78% using additional vacuum treatment under controlled conditions. Bruhin et al. found a comparable closure rate of 72% in a pooled data analysis in non-septic patients using commercial negative pressure wound treatment (NPWT) kits in the open abdomen and 82% by the addition of a ‘dynamic’ closure method [24].

Regardless of the question of vacuum use, the essential importance remains the consistent approximation of the abdominal fascia as soon as the abdominal pressure allows. Consequently, Atema et al. concluded in the review mentioned above that uniform recommendations towards one technique cannot be made at this time [15].

Facing fascia dehiscence following OAT means a substantial problem since stiffness of the fascial edges and close contact of small bowel limit any further surgery. In this series, the standard for recurrent dehiscence was to reexplore the abdominal wall and try to approximate the fascial edges by single stiches using long-time absorbable suture. If there was any evidence that this procedure would fail, an absorbable polyglactine mesh was sutured as inlay technique and the skin was closed by a running suture if there was no obvious evidence of infection.

In this analysis, wound-healing disorders were observed in one-third of the patients. However, wound healing was significantly better in the transverse incision group. The results reflect the problem of wound healing in patients in staged lavage. The underlying mechanism here is not fully understood. It is likely that the thicker tissue coverage and better blood supply to the lateral abdominal wall could cause fewer wound-healing disorders. Nevertheless, the data on this aspect are inconclusive. In a large prospective study, Seiler et al. found more wound infections in transverse incisions [26].

In cases of wound-healing disorder or wound infection without any evidence of fascia dehiscence, NPWT was the treatment of choice.

## Perspective

Late results of laparotomy are not a central aspect of this analysis. However, they should also be taken into account. In this context, incisional hernia is one of the most important late complications [27]. Previous studies have provided evidence that transverse incision can decrease the rate of hernia formation [21], [28].

## Notes

### Competing interests

The authors declare that they have no competing interests.

### Ethical approval

Ethical approval for retrospective analysis was provided by the Ethics Committee of Hamburg Medical Association (Hamburger Ärztekammer), Germany, register no. #WF072/20.

## Figures and Tables

**Table 1:**
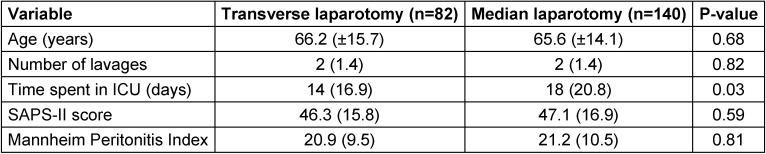
Patient characteristics

**Table 2:**
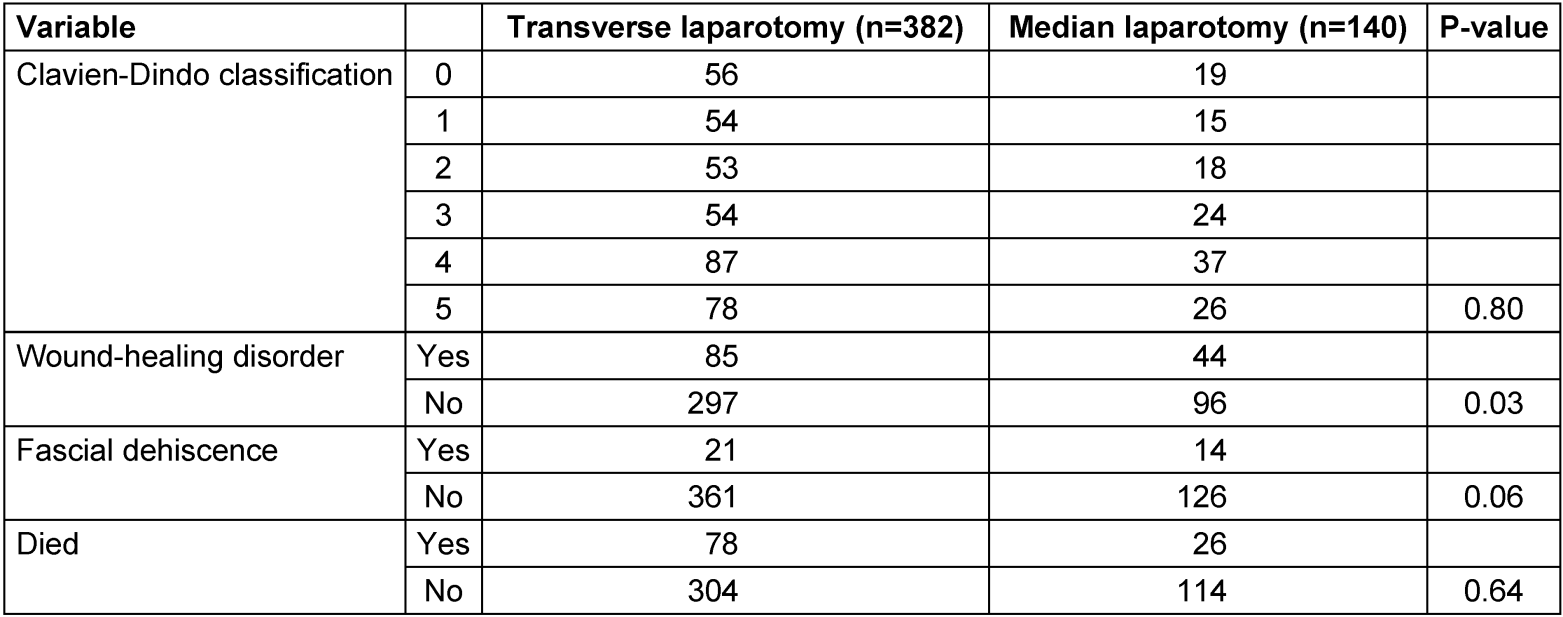
Results according to type of incision
